# Virtual Reality for Anxiety Reduction Demonstrated by Quantitative EEG: A Pilot Study

**DOI:** 10.3389/fpsyg.2018.01280

**Published:** 2018-07-24

**Authors:** Jeff Tarrant, Jeremy Viczko, Hannah Cope

**Affiliations:** ^1^NeuroMeditation Institute, Corvallis, OR, United States; ^2^Department of Psychology, University of Victoria, Victoria, BC, Canada

**Keywords:** virtual reality, VR, Qeeg, sLORETA, mindfulness, anxiety, GAD, nature

## Abstract

While previous research has established that virtual reality (VR) can be successfully used in the treatment of anxiety disorders, including phobias and PTSD, no research has examined changes in brain patterns associated with the use of VR for generalized anxiety management. In the current study, we compared a brief nature-based mindfulness VR experience to a resting control condition on anxious participants. Self-reported anxiety symptoms and resting-state EEG were recorded across intervals containing quiet rest or the VR intervention. EEG activity was analyzed as a function of global power shifts in Alpha and Beta activity, and with sLORETA current source density estimates of cingulate cortex regions of interest. Results demonstrated that both a quiet rest control condition and the VR meditation significantly reduced subjective reports of anxiety and increased Alpha power. However, the VR intervention uniquely resulted in shifting proportional power from higher Beta frequencies into lower Beta frequencies, and significantly reduced broadband Beta activity in the anterior cingulate cortex. These effects are consistent with a physiological reduction of anxiety. This pilot study provides preliminary evidence supporting the therapeutic potential of VR for anxiety management and stress reduction programs.

## Introduction

Anxiety disorders are the most common mental health disorder in the United States. Prevalence estimates from population-based surveys indicate that as much as 1/3 of the population has experienced an anxiety disorder during their lifetime (Bandelow and Michaelis, 2015), resulting in significant loss of productivity, health consequences, and emotional distress (Kessler et al., 2005; Stein et al., 2005; Sareen et al., 2006; Saarni et al., 2007; Bandelow and Michaelis, 2015). Standard treatment options include pharmacotherapy and cognitive-behavioral therapy. While there is good evidence for both of these interventions (Roy-Byrne and Cowley, 2002; Butler et al., 2006; Norton and Price, 2007), it is also clear that only a fraction of those identified as having a diagnosable anxiety disorder receive sufficient treatment (Stein et al., 2011). In addition, there are large numbers of people suffering with undiagnosed anxiety. For example, in a review of epidemiological studies with a total of 48,214 participants, it was found that the prevalence for subthreshold generalized anxiety disorder (GAD) was twice that for the full syndrome (Haller et al., 2014). Studies also indicate that subthreshold GAD tends to be persistent and results in significantly more functional impairment, psychotropic medication use, and accessing of health care services than in non-anxious individuals (Haller et al., 2014).

While cognitive-behavioral therapies (CBT) have been identified as the treatment of choice for GAD, many successful CBT protocols include relaxation training as a central component (Tyrer and Baldwin, 2006). In fact, applied relaxation techniques have been shown to have comparable effects to CBT in short term studies (Siev and Chambless, 2007; Cuijpers et al., 2014). Therapeutic approaches that incorporate mindfulness training also appear to show promise as a treatment for GAD (Wetherell et al., 2011; Hoge et al., 2013).

Given the number of people affected, it seems a logical step to explore the potential of accessible, user-friendly, engaging technologies to assist in this treatment process. This seems particularly relevant for the teaching and provision of applied relaxation and/or mindfulness interventions. Recent evidence suggests that immersive technologies, such as virtual reality (VR), when applied in a specific therapeutic context may an appropriate candidate for this exploration (Maples-Keller et al., 2017).

A review of VR research shows that this modality has been successfully used in the treatment of a variety of anxiety disorders, including phobias and PTSD (Motraghi et al., 2014; Morina et al., 2015). These therapeutic applications have been conducted in the context of an ongoing therapeutic relationship using scenes created in a VR environment, thus becoming sophisticated additions to the traditional model of exposure therapy. In part, the success of these programs appears to be based on the understanding that immersive environments, such as those provided in VR, can generate strong feelings of “presence” (Waterworth et al., 2010; Riva et al., 2011; Riva and Waterworth, 2014; Waterworth and Riva, 2014). “Presence,” in this context, is defined as the subjective feeling of being in another place and is a crucial element in exposure-based therapies. Because VR is immersive, it should not be surprising that this format can provide more presence than 2-dimensional scenes. For example, one recent study found that 360-degree video generated more intense feelings of awe than standard 2-dimensional videos (Chirico et al., 2017).

While our understanding of immersive technologies in the treatment of some anxiety disorders is advancing, only one study to date has explored the use of VR in the treatment of Generalized Anxiety Disorders (GAD). Gorini et al. (2010) compared the impact of using VR with biofeedback, VR without biofeedback, and a wait list control group as an intervention for persons diagnosed with GAD. The VR experiences used in this study were combined with additional therapeutic techniques over the course of 8 weeks. The VR experience itself involved the client exploring a tropical island leading to either a campfire, beach, or waterfall. The experience included an audio narrative of a progressive muscle relaxation technique and/or autogenic techniques. The biofeedback group was able to use heart rate measurements to influence fire intensity, or movement of water. Clients were provided with a simplified version of the experiences using a mobile phone for home practice (Gorini et al., 2010). Pre–post analyses indicated that both experimental groups demonstrated improved clinical outcomes at the end of the treatment period. Physiological measures indicated a tendency toward decreased heart rate and galvanic skin response between the pre- and post-session measurements with the biofeedback group showing slightly larger improvements.

While this study suggests that VR may be a useful tool in the treatment of GAD, it had significant limitations. The sample size of this study was quite small, with one of the treatment groups only having four participants, thus limiting confidence in the results. Additionally, this study included numerous uncontrolled variables, making it difficult to know which aspects of the VR or VR plus biofeedback experience were related to the results. For example, participants focused on various aspects of the VR experience every 2 sessions, making it difficult to know which elements were most effective. In short, much more research is needed in this area.

To examine the potential use of VR as an accessible intervention for generalized anxiety, we thought it was important to examine the impact of a single exposure on anxiety levels. In this study, we examined the impact of a VR experience designed by StoryUp VR which includes several components designed to decrease anxiety. Specifically, the design elements were created based on previous research indicating that both exposure to nature, and mindfulness practices can aid in relaxation and anxiety reduction.

Gorini et al. (2010) used a nature-based VR experience in their study of GAD and obtained positive results. This is consistent with research showing that exposure to nature reliably reduces the stress response. This finding has been reported across multiple studies and was even present when nature was presented in the form of plants, posters, slides, videos, etc. These changes in the stress response have been quantified through a variety of physiological monitoring techniques including muscle tension, skin conductance, pulse transit time, cardiac response, and hormone levels (Berto, 2014). Studies examining EEG changes in response to nature have demonstrated increases in cortical Alpha amplitude (associated with a relaxation response) when viewing slides of natural landscapes versus urban scenes (Ulrich, 1981), when viewing plants with flowers versus pots without flowers (Nakamura and Fujii, 1990), and when watching a green space versus a concrete block fence (Nakamura and Fujii, 1992).

There is also growing evidence that mindfulness-based practices can result in reduced stress and anxiety. In a review and meta-analysis of meditation programs, Goyal et al. (2014) found that mindfulness meditation programs demonstrated moderate evidence as an intervention for anxiety. Perhaps because of this understanding a recent review on the use of VR technology in the treatment of anxiety specifically noted that incorporating mindfulness exercises into a VR experience could be a potentially helpful intervention in the treatment of GAD (Maples-Keller et al., 2017).

The EEG patterns most associated with stress and anxiety are increased fast wave activity (Beta) and decreased slow wave activity (e.g., Alpha; Price and Budzynski, 2009; Olbrich et al., 2011). Thompson and Thompson (2007) noted that anxiety is generally associated with an increase of 19–22 Hz activity found in conjunction with a decrease of 15–18 Hz activity. Obsessive worry is connected to excessive Beta activity along the midline and at electrode site Cz (Hammond, 2005b). Using LORETA (Low Resolution Electromagnetic Tomogrophy) analyses, Sherlin (2009) indicated that the most common pattern associated with anxiety is excessive Beta in the anterior cingulate or midline cortex.

As noted by Sherlin and Wyckoff (2010), it is logical to assume that the same brain regions aroused by anxiety would show quieting patterns during relaxation and meditation. In fact, one of the most common forms of neurofeedback for anxiety treatment involves Alpha training (Moore, 2000; Hammond, 2005a,b). Increasing Alpha tends to result in subduing higher frequency activity and cortical overexcitability across the cortex, much the way training to increase Beta results in increases in higher frequency cortical activation. These patterns are seen in other research relevant to the current study. For example, increases in Alpha power are associated with lower levels of anxiety, increased calmness, positive affect, and a range of other autonomic changes associated with decreased sympathetic arousal (Cahn and Polich, 2006).

Multiple studies have identified specific regions of the brain associated with stress and anxiety. Most notably, the cingulate gyrus is thought to play a significant role in the regulation of nervous system arousal (Critchley et al., 2003). The cingulate gyrus runs down the midline of the brain, immediately superior to the corpus collosum. This region has been shown to become activated during the experience of pain (Vogt et al., 1996), negative emotions and memories (Maddock et al., 2003a,b), and anxiety (Fredrikson et al., 1997; Simpson et al., 2001; Lanius et al., 2003). To examine this region, we used sLORETA (Pascual-Marqui, 2002) analyses to examine current source density (CSD) estimates at the anterior and posterior cingulate as these specific areas contribute important and distinct elements to the experience of anxiety.

The current study investigated the impact of a mindfulness-in-nature VR intervention on persons screened as demonstrating moderate to high levels of generalized anxiety. Changes in anxiety were assessed through self-report questionnaires (STAI-state) as well as EEG patterns associated with anxiety and/or relaxation. We employed a mixed model repeated-measure approach, whereby EEG recordings and state anxiety ratings were made across three time points spanning two 5-min intervals, either containing the meditative VR experience or 5 min of awake, eyes-open rest. For the intervention group the measurement and interval sequencing was comprised of an initial EEG baseline, followed by rest, followed by post-rest EEG and state anxiety measurements, followed by the interval of the VR meditation experience, followed by a final EEG recording and state anxiety measurement. The control group followed the same recording procedures and intervals, with the exception that they participated in a further interval of quiet rest, rather than the VR meditation. We hypothesized that the meditative VR experience, more than rest alone, would result in a significant reduction in reported state levels of anxiety. Furthermore, we hypothesized that such reductions in state anxiety would be accompanied by equally significant changes in overall EEG activity. More specifically, we anticipated a significant drop in anxiety to be associated with a reduction in the amount of high frequency activity in favor of power in lower frequency ranges.

## Materials and Methods

### Participants

Participants were recruited in two separate rounds through flyers and marketing on Facebook^®^. The initial subject pool were all part of the intervention group. Following preliminary analyses, it was determined that a control group was necessary to better interpret the results. Consequently, the same procedures were repeated for a second group of participants that served as the control group.

Interested participants called the researcher and completed a phone screening consisting of exclusion/inclusion criterion as well as a Generalized Anxiety Disorder screening (GAD-7). Exclusion criterion included a history of head injury, seizure activity, or major mental health concerns (schizophrenia and bipolar disorder). To be included in the study, participants had to be at least 18 years old with a moderate level of generalized anxiety (score of 8 or higher on the GAD-7. Intervention *M* = 12.6, *SD* = 3.7; Control *M* = 12.0, *SD* = 4.14). A total of 47 respondents were phone screened in the intervention group, 26 were eligible to participate and 21 completed the study. Of the five that did not complete the study, three canceled and two did not show for their appointments. The complete data set of seven participants were removed due to excessive EEG artifact or incomplete data sets due to researcher error (*N* = 14). Eighteen respondents were screened for the control group, 5 were not qualified due to GAD scores lower than 8 and one of those five had a traumatic brain injury. Of the 13 controls subjects that completed the study, one was removed due to excessive artifact (*N* = 12). For demographic information and participants’ previous experience with meditative practices, see **Table 1**. The study was performed at the NeuroMeditation Institute, LLC in Corvallis, OR, United States. It was approved by the Quorum Institutional Review Board, Seattle, WA, United States.

**Table 1:** Demographic characteristics and previous meditative experience between groups.

	EX (*n* = 14)		CN (*n* = 12)	
Age				
Mean	46.21		48.17	
Standard deviation	10.77		20.11	
Gender	#	*%*	#	*%*
Female	11	*78.6%*	9	*75.0%*
Male	3	*21.4%*	3	*25.0%*
Race/ethnicity				
Caucasian	12	*85.7%*	10	*83.3%*
Asian/Pacific Islander	0		1	*8.3%*
Multi-Racial	0		1	*8.3%*
Other	2	*14.3%*	0	
Education level				
High school	0		4	*33.3%*
Some college	5	*35.7%*	1	*8.3%*
Associate’s degree	1	*7.1%*	0	
Bachelor’s degree	4	*28.6%*	3	*25.0%*
Master’s degree	3	*21.4%*	4	*33.3%*
Doctoral Degree	1	*7.1%*	0	
Meditation practice^a^ past 6 months (sessions/week)				
None	6	*42.9%*	5	*41.7%*
1–2	3	*21.4%*	4	*33.3%*
3–4	2	*14.3%*	1	*8.3%*
5–6	0		1	*8.3%*
6+	3	*21.4%*	1	*8.3%*
Weekly time spent in meditation (mins)				
0	6	*42.9%*	5	*41.7%*
1–60	6	*42.9%*	6	*50.0%*
60–120	2	*14.3%*	0	
120–180	0		0	
180–240	0		1	8.3%
240+	0		0	


*^a^Examples of meditative practices in questionnaire included: seated meditation, yoga, qigong, chanting, prayer or other practices. EX, Participants in VR meditation experience group; CN, Resting control group.*

### Measures

#### Demographic Questionnaire

This questionnaire asked subjects to identify information related to their sex, age, race, education level, experience with meditative practices and history of mental illness.

#### Generalized Anxiety Disorder-7 (GAD-7)

This 7-item self-report scale asks subjects to identify how much they were bothered by each of 7 symptoms during the previous 2 weeks. Response options include, “not at all,” “several days,” “more than half the days,” and “nearly every day,” scored 0, 1, 2, and 3, respectively. Total score ranges from 0 to 21 with higher scores indicating higher anxiety and a higher likelihood of meeting criterion for GAD. A score of 8 has been shown to have a 92% sensitivity and a 76% specificity in relation to a diagnosis of GAD (Spitzer et al., 2006). This was the cut-off utilized in the current study. In addition, the GAD-7 has previously demonstrated an internal consistency of 0.92 and test-retest reliability of 0.83 (Spitzer et al., 2006).

#### State-Trait Anxiety Inventory-Y (STAI)

The STAI is a commonly used measure of trait and state anxiety. Form Y has 20 items for assessing trait anxiety and 20 for state anxiety. Only the state portion of the survey was used in this study as this segment was designed to measure more immediate symptoms of anxiety. State items include: “I am tense;” “I am worried;” and “I feel calm.” All items are rated on a 4-point scale (e.g., from “not at all” to “very much so”). Higher scores indicate greater anxiety. Internal consistency coefficients for the scale have ranged from 0.86 to 0.95; test–retest reliability estimates have ranged from 0.65 to 0.75 over a 2-month interval (Spielberger et al., 1983).

#### EEG Data Collection

**FIGURE 1:**
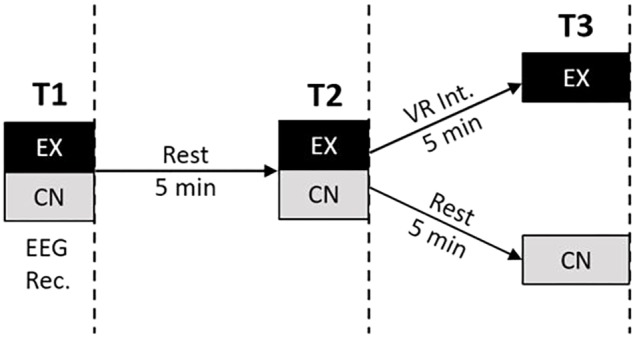
Experimental design. Participants were recruited to either the VR experience group (EX) or a control group (CN). Each group underwent three time points of measurement each consisting of 5-min eyes closed EEG recording and filling out state anxiety questionnaire. EEG recordings were interleaved by an initial 5 min period of eyes-open rest for both groups, followed by either a brief 5 min VR meditation intervention (EX) or another period of 5 min eyes-open rest (CN).

The EEG data in this study was sampled with 19 electrodes in the standard 10–20 International placement referenced to linked ears. Electrode sites corresponded to Fp1, Fp2, F3, F4, F7, F8, Fz, C3, C4, Cz, P3, P4, Pz, T3, T4, T5, T6, O1, and O2. **Figure 1** illustrates the experimental design and EEG recording intervals. While all the rest intervals were of quiet, eyes-open rest, the EEG-recordings were conducted during eyes-closed resting. Five minutes of eyes-closed data resting was collected at three recording time points for each subject: Time 1 (Baseline), Time 2 (2nd Baseline), Time 3 (Post Experimental Condition). Between the recordings, for Time 1 and Time 2, all subjects were instructed to sit in a state of quiet, natural, eyes-open rest (blinking allowed) for 5-min. This served as a within-subjects control condition for the intervention group. Each raw EEG file was uploaded to Qeeg Pro (QEEG Professionals, The Netherlands) and processed through a Standardized Artifact Rejection Algorithm (S.A.R.A). This process removes segments from an EEG recording that are likely due to other sources, such as eye blinks, muscle tension, etc. Using an automated process such as this ensures that each file is handled in the same manner and reduces the possibility of bias in the artifact removal process. Raw files were then manually inspected. 11.5% of the experimental group and 7.7% of the control group EEG recordings were eliminated due to excessive artifact.

Artifact-free files were then processed through BrainAvatar software (BrainMaster Technologies, Inc.) to obtain power estimates for the bands Alpha (8–12 Hz), Alpha1 (8–10 Hz) and Alpha2 (10–12 Hz) sub-bands, Beta (12–30 Hz), Low Beta (12–18 Hz), and High Beta (18–30 Hz) sub-bands, and broadband activity (Sum: 1–30 Hz) at all 19 electrode sites. In addition, CSD estimates for the same primary EEG bands were obtained for specific regions of interest (ROI’s) using the sLORETA algorithm (Pascual-Marqui, 2002) in the BrainAvatar software (BrainMaster Technologies, Inc.). sLORETA is the standardized version of LORETA (Low Resolution Electromagnetic Tomographic Analysis). Both LORETA and sLORETA generate solutions to the “inverse” problem of EEG electrophysiology by using a set of scalp EEG measurements to produce estimates of activity measured in CSD. While the accuracy of CSD estimates increases with the number of electrodes (Song et al., 2015), it has been shown that with as little as 16 electrodes (Cohen et al., 1990), and using the approximate three-shell head model provided by the original LORETA equations, “human *in vivo* localization accuracy of EEG is 10 mm at worst” (Pascual-Marqui, 1999, p. 11). Given that sLORETA provides higher resolution than the previous solution (LORETA), accuracy of localization should be improved (Pascual-Marqui, 2002) and sufficient for the current pilot examination. The specific application utilized in this study uses 5-mm voxels to compute 6,239 voxels which can be combined to correspond to 88 specific brain regions (Collura, 2012).

### Procedures

Importantly, for testing the VR intervention, our initial study approach was a within-subject, rest-then-intervention, experimental design. After more resources became available, the participants for the control group were then recruited, screened, and ran, allowing for within- and between-group comparisons. However, the sequential addition of this more rigorous control method resulted in non-random assignment to groups. Although participants in both groups matched closely on all demographic characteristics (see **Table 1**), this is still a notable procedural limitation.

Participating subjects were scheduled for a 75-min office visit. After a verbal description of the study process and completing IRB information/consent forms, subjects completed a demographic, and STAI-state questionnaire. Subjects were then provided with a non-therapeutic VR experience to orient them to the experience of VR. During the VR orientation, subjects were seated in a swivel chair in the center of the research room, given basic instruction on the VR headgear (Gear VR powered by a Samsung Android s7 phone), and then given the opportunity to experience a 2 min, 30 s VR event that involved being on the field just prior to the beginning of a college football game. Following this orientation, subjects were fitted with a 19-channel EEG electrocap (Electrocap International, United States). Each electrode was prepped using electrogel conductance paste (Electro-Cap International, Inc., United States). Impedances for all sites were assessed prior to each recording and kept below 10 kOhms. Subjects completed a 5-min, eyes-closed EEG baseline, recorded using a BrainMaster Discovery amplifier (BrainMaster Technologies, Inc., United States). Following the initial baseline recording, subjects were asked to remain seated with eyes open and no verbal interaction for 5 min. After this resting period, a second eyes closed baseline was recorded for 5 min and subjects were asked to complete a second STAI-state questionnaire. This process provided a within-subject control, allowing us to investigate whether the VR experience results in significantly more change than might be observed simply by time. Control group subjects repeated a second 5 min, eyes-open resting period while experimental group subjects returned to the swivel chair and the VR headgear was placed over the electrocap. The subject was then instructed to simply enjoy the mindfulness in nature VR experience and follow along with the guided meditation.

**FIGURE 2:**
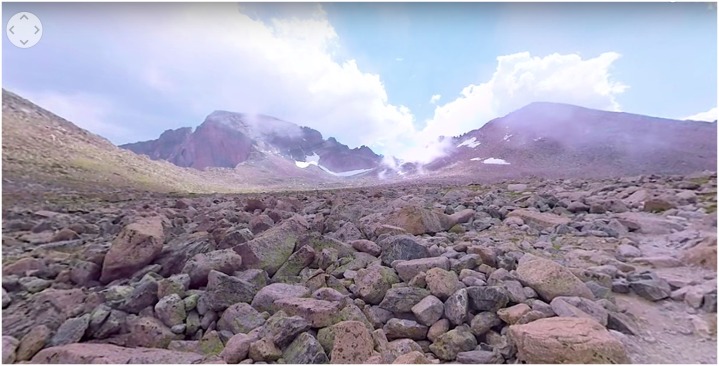
Mindfulness in nature VR environment.

The Mindfulness in nature experience was 5-min, 41 s in length and produced by StoryUp VR (Columbia, MO, United States) using 360° video photography. As the scene opens, there are mountains in the distance and large rocks all around on the landscape (see **Figure 2**). The sky is blue and speckled with clouds and there is a mist rolling in front of the mountains. There is soft piano and violin music playing in the background. Approximately 20 s into the experience, a woman’s voice begins guiding the viewer through a mindfulness meditation, directing the attention to elements of the environment and asking them to connect with what they are seeing by imagining that they embody the same qualities as the rocks and sky. Near the end of the scene, an inspirational quote attributed to Lao Tzu (i.e., “Nature does not hurry, yet everything is accomplished”) is displayed and the screen slowly dims.

At the conclusion of the second rest period (control group) or the mindfulness-in-nature VR experience (intervention group), the headgear was removed, impedance levels were re-checked, and a post-VR, 5-min EEG was recorded using the same instructions as the previous recordings. Following the final EEG recording, subjects completed a post-VR STAI-state form questionnaire. All subjects completing the study were incentivized for their participation with a $25 gift card.

### Data Analysis

Both mean power and power band ratios from the average of all 19 electrodes were used to test hypotheses about global spectral state changes across time points. As a pilot study our primary aim was to capture the spectral changes broadly and not tightly coupled to topographical restrictions. We anticipated the whole scalp average approach would be able to capture the predicted changes in the Alpha and Beta bands, and also limit the number of statistical comparisons employed to test our hypotheses. As part of our analyses we also aimed to analyze relative spectral power shifts in participant EEG profiles between power bands, so simple power ratios were also calculated from the total electrode power means. Ratios of interest included each band and sub-band power over Sum power (1–30 Hz) as well as Alpha/Beta, Alpha1/Alpha2, and Low-Beta/High-Beta.

For increased specificity, we divided Alpha (8–12 Hz) and Beta (12–30 Hz) into two sub-components. Alpha1 (8–10 Hz), sometimes referred to as “Low Alpha,” is associated with a calm and relaxing state in which we are not attending to the external world (Thompson and Thompson, 2003). In contrast, Alpha2 (10–12 Hz), sometimes referred to as “High Alpha,” is related to a state of relaxed alertness, such as might be observed just prior to engaging in an action (Thompson and Thompson, 2003). The lower end of Beta (12–18 Hz) activity has been implicated in, and emerging from, the undertaking challenging cognitive tasks (Sherlin, 2009). It has also been associated with the maintenance of cognitive, psychological, and behavioral processes, with pathological elevations in Beta activity potentially linked to states of cognitive inflexibility (Engel and Fries, 2010). High Beta (18–30 Hz) is most often associated with higher levels of concentration, but is also observed at increased levels during anxiety or periods of emotional intensity (Thompson and Thompson, 2003; Franken et al., 2004; Sherlin, 2009).

Our inclusion of sLORETA ROIs for analysis was also restricted for minimum variable inclusion. We limited the analyses to two ROIs: the Posterior Cingulate Cortex (PCC), a major hub of the default mode network (Shulman et al., 1997; Raichle et al., 2001), and the Anterior Cingulate Cortex (ACC), an area associated with cognitive and emotional processing (Bush et al., 2000; Holroyd and Coles, 2002; Mansouri et al., 2009). Both regions have also been associated with state changes during, and as a result of meditative experiences (Holzel et al., 2011; van Lutterveld et al., 2016).

Statistical analyses were performed with SPSS software (SPSS v21; IBM Inc., Armonk, NY, United States). A series of 2 × 3 (Group × Time) mixed model repeated measure ANOVAs were computed for each EEG frequency band of interest, for both mean power and band power ratios. EEG activity was computed across all 19 electrode sites to incorporate the most EEG data as possible and evaluate changes at the broadest scale. Similarly, 2 × 3 (Group × Time) mixed-model repeated-measure ANOVAs were also used to analyze the sLORETA estimated current source densities of ACC and PCC activity in the Theta, Alpha and Beta range. However, here we limited analyses to the broad band level (i.e., Theta, Alpha, Beta), and did not include sub-band computations. Huynh–Feldt corrections to degrees of freedom were applied when violations to variable sphericity across time points were detected. The threshold for evaluating significance was set to α = 0.05. Follow-up one-way ANOVAs were conducted when group or interaction differences were detected, followed by pairwise comparisons, Bonferroni corrected for multiple comparisons.

Of the 34 total participants, two participant’s data (one from each group) was removed because of high levels of artifact EEG contamination across all time points. In addition, 6 subjects were missing at least some data in a specific recording condition. For the final analyses, only the subjects with full data sets were used. Thus, the final sample (*N* = 26) was comprised of 14 participants in the VR group and 12 participants in the control group.

## Results

### Demographic

Control group data was collected after intervention group data due to previously noted constraints. Because these circumstances precluded random assignment between groups, we attempted to match the subsequent control group to the screening and demographic characteristics of the previously collected intervention group. Statistical analyses with independent *t-*testing, revealed no statistical differences in terms of age, years of education, GAD, or meditation experience. **Table 1** reveals the comparability between groups across all demographic variables.

### State-Trait Anxiety Inventory

To evaluate subject reports of state stress levels across time points, mean STAI scores were analyzed using a repeated-measures 2 × 3 (Group × Time) ANOVA. A significant main effect for Time was observed, *F*_1.4,32.7_ = 35.54, *p* < 0.001, $\eta_{p}^{2}$ = 0.60, with no between Group or interaction effects (Group: *F*_1,24_ = 1.72, *p* = 0.202, $\eta_{p}^{2}$ = 0.07; Group × Time: *F*_1.4,32.7_ = 0.54, *p* = 0.522, $\eta_{p}^{2}$ = 0.02). State stress ratings linearly declined across each interval for both groups, regardless of whether intervals contained rest or the intervention. Means and standard errors across time points for STAI respective to group, and all other dependent variables are contained in **Table 2**.

**Table 2:** Means and standard errors for anxiety ratings and EEG power.

	*T1*				*T2*				*T3*			
			
	EX		CN		EX		CN		EX		CN	
Anxiety Rating					
STAI	2.339	*0.118*	2.125	*0.127*	2.014	*0.122*	1.752	*0.132*	1.682	*0.111*	1.567	*0.120*
Absolute Power												
Alpha	5.774	*0.667*	3.819	*0.721*	6.115	*0.694*	3.846	*0.749*	6.355	*0.673*	4.110	*0.727*
Beta	4.991	*0.355*	4.006	*0.383*	5.203	*0.381*	4.083	*0.411*	5.280	*0.366*	4.150	*0.395*
Alpha1	4.298	*0.617*	2.948	*0.666*	4.498	*0.593*	2.929	*0.641*	4.832	*0.606*	3.150	*0.655*
Alpha2	3.612	*0.463*	2.333	*0.500*	3.910	*0.522*	2.425	*0.563*	3.892	*0.489*	2.575	*0.528*
Low-Beta	3.320	*0.246*	2.514	*0.266*	3.434	*0.266*	2.584	*0.287*	3.552	*0.261*	2.631	*0.282*
High-Beta	2.976	*0.206*	2.709	*0.223*	3.082	*0.211*	2.754	*0.228*	3.049	*0.202*	2.763	*0.218*


*Anxiety ratings correspond to the mean score across participants on the 20 items State-Trait Anxiety Index (STAI) specifically evaluating current state anxiety. Units are in microvolts-squared (μV^2^) for mean power. Standard error of mean values are italicized.*

### Mean Power

To evaluate the electrophysiological markers of cognitive state change across intervals we first analyzed the mean power from all electrode sites for each frequency band of interest. Alpha (*F*_2,48_ = 7.06, *p* = 0.002, $\eta_{p}^{2}$ = 0.23) and Beta (*F*_2,48_ = 5.84, *p* = 0.005, $\eta_{p}^{2}$ = 0.20) demonstrated a significant main effect for time, with both groups demonstrating slight linear increases in power across intervals. When both Alpha and Beta were broken down into their sub-bands for increased resolution both Alpha1 (*F*_2,48_ = 6.11, *p* = 0.004, $\eta_{p}^{2}$ = 0.20) and Alpha2 (*F*_2,48_ = 5.75, *p* = 0.006, bands for increased resolution both Alpha = 0.004), as well as Low Beta (*F*_2,48_ = 8.92, *p* = 0.001, $\eta_{p}^{2}$ = 0.26), but not High Beta (*F*_2,48_ = 2.59, *p* = 0.085, $\eta_{p}^{2}$ = 0.098), revealed significant main effect for time. Across intervals, Alpha1 power increased for both groups, but more so for the intervention group after VR. In contrast, for both groups across rest Alpha2 power shows a small increase, which continues after the second period of rest for the control group, but demonstrates a slight decrease after the VR experience for the intervention group. Group means and standard errors of mean power for each group can be seen in **Table 2**, and reveal comparable linear decreases across all intervals for both groups.

No significant time by group interactions were observed for Alpha, Beta, or the respective sub-band activity of each. However, a significant main effect of group difference was observed for both Alpha (*F*_1,24_ = 4.76, *p* = 0.039, $\eta_{p}^{2}$ = 0.17) and Low Beta (*F*_1,24_ = 5.21, *p* = 0.032, $\eta_{p}^{2}$ = 0.18), to the effect that on average the intervention group had higher power than the control group. Closer investigation of these differences with follow-up one-way ANOVAs at each time point revealed for Alpha initially the intervention and control groups were statistically comparable (T1: *F*_1,25_ = 3.97, *p* = 0.058), before becoming increasingly different across subsequent intervals (T2: *F*_1,25_ = 4.94, *p* = 0.036; T3: *F*_1,25_ = 5.14, *p* = 0.033). Low Beta was found to be initially significantly higher for the intervention group (T1: *F*_1,25_ = 4.96, *p* = 0.036; T2: *F*_1,25_ = 4.72, *p* = 0.040), and similarly to Alpha, was most different between groups at the last recording time (T3: *F*_1,25_ = 5.74, *p* = 0.025). In both cases, the intervention group started with higher average Alpha and Low-Beta spectral power, with the pattern of results showing further power increases particularly for the intervention group after the VR meditation experience (Alpha: T_1-2_ *M_±SE_* = -0.027_±.125_, *p* = 0.998, T_2-3_ *M_±SE_* = -0.264_±.147_, *p* = 0.302; Low Beta: T_1-2_ *M _±SE_* = -0.114_±0.083_, *p* = 0.582, T_2-3_ *M _±SE_* = -0.232_±0.073_, *p* = 0.021) compared to the control group after their second period of quiet rest (Control Alpha: T_1-2_ *M_±SE_* = -0.341_±0.207_, *p* = 0.369, T_2-3_ *M_±SE_* = -0.240_±.111_, *p* = 0.149; Low Beta: T_1-2_ *M_±SE_* = -0.070_±0.035_, *p* = 0.205, T_2-3_ *M_±SE_* = -0.047_±0.052_, *p* = 0.137). This difference likely contributed to the overall group difference effect found in Alpha and Low Beta revealed by the initial omnibus ANOVA.

Together these results reveal significant increases in Alpha and Beta power over the study duration, with the power in the lower, but not higher, Alpha and Beta sub-bands demonstrating slightly higher power increases on average specifically after the VR intervention as opposed to rest.

### Power Ratios

To further investigate spectral power shifts from the perspective of relative power changes, we converted the mean power into ratio form for comparison. We looked at the specific power dynamic changes on the backdrop of global power (i.e., Sum; 1–30 Hz), as well as between specific band and sub-band frequency ranges. A significant main effect for time was observed for Alpha/Sum (*F*_2,48_ = 4.57, *p* = 0.015, $\eta_{p}^{2}$ = 0.16), and Alpha/Beta (*F*_2,48_ = 5.26, *p* = 0.009, $\eta_{p}^{2}$ = 0.18), but not Beta/Sum (*F*_2,48_ = 0.67, *p* = 0.523, $\eta_{p}^{2}$ = 0.03). Overall alpha increased in proportional power over Sum and Beta activity over the course of the experiment for both groups, whereas Beta/Sum tended toward a slight, non-significant decrease across the experimental intervals. Ratio means for band and sub-band dynamics can be viewed in **Table 3**. No significant group differences or interaction effects were observed for Alpha/Sum, Alpha/Beta, or Beta/Sum.

**Table 3:** Means and standard errors for power ratios.

	T1				T2				T3			
			
Power Ratio	EX		CN		EX		CN		EX		CN	
Alpha/Sum	0.612	*0.039*	0.541	*0.042*	0.618	*0.037*	0.538	*0.040*	0.633	*0.033*	0.568	*0.035*
Beta/Sum	0.569	*0.018*	0.586	*0.020*	0.568	*0.019*	0.582	*0.021*	0.563	*0.017*	0.583	*0.019*
Alpha/Beta	1.097	*0.094*	0.960	*0.101*	1.112	*0.088*	0.952	*0.095*	1.146	*0.084*	1.003	*0.090*
Alpha1/Sum	0.451	*0.044*	0.421	*0.047*	0.457	*0.039*	0.412	*0.042*	0.482	*0.038*	0.437	*0.041*
Alpha2/Sum	0.389	*0.035*	0.327	*0.038*	0.396	*0.035*	0.337	*0.038*	0.391	*0.033*	0.354	*0.035*
High-Beta/Sum	0.352	*0.024*	0.400	*0.025*	0.351	*0.024*	0.396	*0.026*	0.339	*0.022*	0.390	*0.024*
Low-Beta/Sum	0.378	*0.015*	0.369	*0.017*	0.374	*0.015*	0.368	*0.016*	0.378	*0.015*	0.370	*0.016*
High-Beta/Alpha	0.634	*0.087*	0.826	*0.094*	0.622	*0.081*	0.800	*0.087*	0.579	*0.071*	0.738	*0.077*
Low-Beta/Alpha	0.656	*0.060*	0.743	*0.065*	0.641	*0.053*	0.729	*0.058*	0.623	*0.047*	0.685	*0.051*
Low-Beta/High-Beta	1.095	*0.046*	0.957	*0.050*	1.088	*0.046*	0.968	*0.050*	1.142	*0.051*	0.980	*0.055*
Alpha1/Alpha2	1.367	*0.187*	1.360	*0.202*	1.342	*0.169*	1.295	*0.183*	1.442	*0.179*	1.302	*0.194*


*Standard error of mean values are italicized.*

**FIGURE 3:**
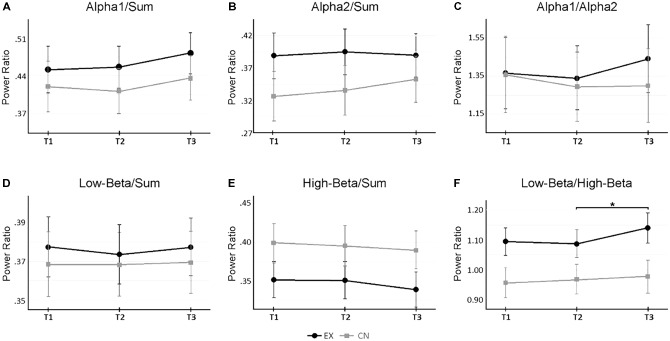
Changes in sub-band power ratios across rest and virtual reality intervals. **(A,B)** Change in Alpha 1 and Alpha2 activity relative to total broadband power (‘Sum’) across measurement time points. **(C)** Relative power changes between Alpha 1 and Alpha2. **(D,E)** Change in Low- and High-Beta activity relative to total broadband power. **(F)** Relative power changes between Low-Beta and High-Beta. A significant interaction *(p <* 0.05) occurred for Low-Beta/High-Beta. ^∗^Denotes *p <* 0.05 for follow up within-subject repeated measure ANOVAs by group, corrected for multiple pairwise comparisons. Alpha 1 = 8–10 Hz, Alpha2 = 10–12 Hz, Low-Beta = 12–18 Hz, High-Beta = 18–30 Hz, Sum = 1–30 Hz.

At sub-band resolution, Alpha1/Sum (*F*_2,48_ = 4.00, *p* = 0.025, $\eta_{p}^{2}$ = 0.14), High-Beta/Sum (*F*_2,48_ = 5.81, *p* = 0.009, $\eta_{p}^{2}$ = 0.18), High-Beta/Alpha (*F*_2,48_ = 9.24, *p* < 0.001, $\eta_{p}^{2}$ = 0.28), Low-Beta/Alpha (*F*_2,48_ = 5.34, *p* = 0.008, $\eta_{p}^{2}$ = 0.18), and Low-Beta/High-Beta (*F*_2,48_ = 13.20, *p* < 0.001, $\eta_{p}^{2}$ = 0.36) all revealed significant main effects across time, with no significant differences between groups. Overall, particular to the second interval, and irrespective of group, Alpha1/Sum significantly increased (T_2-3_ *M_±SE_* = -0.025_±0.009_, *p* = 0.031), whereas Low-Beta/Alpha (T_2-3_ *M_±SE_* = 0.031_±0.011_, *p* = 0.028) and High-Beta/Alpha (T_2-3_*M_±SE_* = 0.053_±0.014_, *p* = 0.002) decreased. High-Beta/Sum (T_1-3_ *M_±SE_* = 0.011_±0.003_, *p* = 0.009) demonstrated a slight linear decrease across the experiment, more pronounced for the intervention group after the VR experience. Alpha2/Sum (*F*_2,48_ = 2.49, *p* = 0.093, $\eta_{p}^{2}$ = 0.09), Low-Beta/Sum (*F*_2,48_ = 0.42, *p* = 0.660, $\eta_{p}^{2}$ = 0.02), Alpha1/Alpha2 (*F*_2,48_ = 1.00, *p* = 0.375, $\eta_{p}^{2}$ = 0.04) did not change significantly across time, or differ significantly between groups. **Figure 3** shows the relative sub-band power dynamics across intervals, by group.

Notably, a significant interaction was observed for Low-Beta/High-Beta (*F*_2,48_ = 3.71, *p* = 0.032, $\eta_{p}^{2}$ = 0.13). Follow-up ANOVA tests revealed that across the first two recording time points, interleaved by a period of rest for both groups, each group’s Low-Beta/High-Beta power was statistically comparable (T1: *F*_1,25_ = 4.15, *p* = 0.053; T2: *F*_1,25_ = 3.14, *p* = 0.089). However, at the last recording, a significant difference was observed (T3: *F*_1,25_ = 4.15, *p* = 0.040). Looking at the within group patterns for the intervention group, there was no significant change in Low-Beta/High-Beta across the rest interval (T_1-2_ *M_±SE_* = 0.007_±.010_, *p* = 0.998), and the significant change only occured after the VR meditation experience (T_2-3_ *M_±SE_* = -0.054_±.014_, *p* = 0.005). This pattern was not observed across either of rest intervals for the control group (T_1-2_ *M_±SE_* = -0.011_±0.008_, *p* = 0.578; T_2-3_ *M_±SE_* = -0.012_±0.008_, *p* = 0.463). **Figure 3F**, illustrates this interaction. These results indicate that the ratio of Low Beta to High Beta increased to favor Low Beta power, as a specific result of the VR experience.

### sLORETA

**FIGURE 4:**
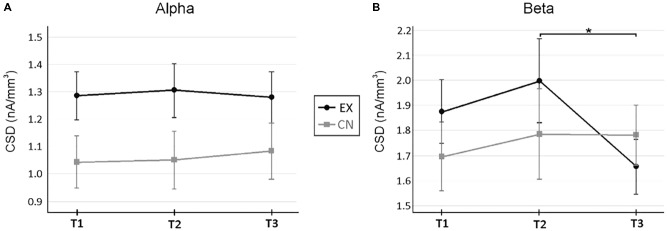
Current source density changes in the anterior cingulate cortex (ACC) across rest and virtual reality intervals. **(A)** Changes in Alpha across measurement time points. **(B)** Changes in Beta activity across measurement time points. A significant interaction *(p <* 0.05) occurred for ACC Beta activity. ^∗^Denotes *p <* 0.05 for follow up within-subject repeated measure ANOVAs by group, corrected for multiple pairwise comparisons. ^∗^Denotes *p* < 0.05, for within-subject repeated measure ANOVA, after correction for multiple pairwise comparisons. Current source densities were estimated with sLORETA (Pascual-Marqui, 2002).

Next, we wanted to investigate specific anterior and posterior cortical regions of interest for changes in regional activity across spectral bands. Specifically, we investigated current source densities in the ACC and PCC for Theta, Alpha, and Beta frequencies. In the ACC, a significant main effect for time was found for Beta (*F*_2,48_ = 3.01, *p* = 0.054, $\eta_{p}^{2}$ = 0.11) but not Theta (*F*_2,48_ = 0.41, *p* = 0.664, $\eta_{p}^{2}$ = 0.02) or Alpha (*F*_2,48_ = 0.47, *p* = 0.649, $\eta_{p}^{2}$ = 0.02). There was no between group main effect for ACC activity, and Theta and Alpha appeared to be quite stable across intervals for both the experimental and control groups across intervals. However, a significant Time by Group interaction emerged for ACC Beta activity (*F*_2,48_ = 3.52, *p* = 0.038, $\eta_{p}^{2}$ = 0.13). Follow-up repeated measures by group indicated no significant change across rest for the experimental group (T_1-2_*M_±SE_* = -0.122_±0.105_, *p* = 0.795), followed by a significant decrease in ACC Beta activity following the VR intervention (T_2-3_ *M_±SE_* = 0.341_±0.010_, *p* = 0.048). The control group did not demonstrate a significant change across either rest interval (T_1-2_ *M_±SE_* = -0.090_±0.042_, *p* = 0.171; T_2-3_ *M_±SE_* = 0.005_±0.098_, *p* = 0.998). Alpha and Beta ACC activity between groups can be found in **Figure 4**.

For the PCC, significant main effects for time were found for Alpha (*F*_2,48_ = 4.42, *p* = 0.017, $\eta_{p}^{2}$ = 0.16) and Beta (*F*_2,48_ = 5.61, *p* = 0.006, $\eta_{p}^{2}$ = 0.19), but not Theta (*F*_1.7,41.8_ = 2.44, *p* = 0.106, $\eta_{p}^{2}$ = 0.09). Significant increases, irrespective of group and interval, were only observed comparing between the first and last time points (PCC Alpha: T_1-3_*M_±SE_* = -0.228_±0.086_, *p* = 0.042; PCC Beta: T_1-3_ *M_±SE_* = -0.122_±0.039_, *p* = 0.015). The experimental group demonstrated consistently higher PCC current source densities than the control group (Alpha: *F*_1,24_ = 4.47, *p* = 0.045, $\eta_{p}^{2}$ = 0.16; Beta: *F*_1,24_ = 4.32, *p* = 0.049, $\eta_{p}^{2}$ = 0.15), but both groups demonstrated the same small pattern of linear CSD increase over time.

Together these results reveal a significant specific effect on ACC Beta activity occurring after the VR experience, but not after rest alone. Alpha activity was uniformly higher in PCC for the VR group across all intervals, demonstrating similar incremental increases across the VR interval as rest. It is unclear what accounts for the group differences in PCC Alpha, however, it does not appear to be directly related to undergoing the VR meditation experience.

#### Correlations

To further quantify the relationship between self-reported anxiety and changes in electrophysiological profiles we conducted correlational analyses examining both STAI and GAD scores in relation to our EEG power ratio and source localization data (*N* = 26 for correlational analyses). GAD scores were significantly inversely correlated with Alpha/Sum across all time points (T_1_, *r* = -0.47, *p* = 0.019; T_2_, *r* = -0.35, *p* = 0.080; T_3_, *r* = -0.43, *p* = 0.027). At the sub-band level it was discovered that there was a proportional increase of slower sub-band Alpha activity (Alpha1/Sum: T_1_, *r* = -0.46, *p* = 0.018; T_2_, *r* = -0.35, *p* = 0.078; T_3_, *r* = -0.43, *p* = 0.029) but not higher Alpha sub-band activity (Alpha2/Sum: T_1-3_, *r*’s = -0.12 – -0.13, *p*’s > 0.2) that supported this relationship. Additionally, and perhaps more germane to our main findings, across all time points Low-Beta/High-Beta was similarly significantly inversely related to GAD scores (T_1_, *r* = -0.45, *p* = 0.023; T_2_, *r* = -0.43, *p* = 0.030; T_3_, *r* = -0.39, *p* = 0.048). Conversely, High-Beta/Sum activity was significantly positively associated with GAD across all time points (*r* = 0.48, *p* = 0.013; *r* = 0.45, *p* = 0.022; *r* = 0.44, *p* = 0.025) whereas low Beta/Sum was not (T_1-3_ *r*’s = 0.10 – 0.12 *p*’s > 0.5).

To analyze the STAI ratings, difference scores were calculated between T2-1, T3-2, and T3-1. Difference scores were also calculated for Alpha, Beta, and their sub-bands across a number of variables. The only positive correlation between STAI and EEG was between T3-2 for mean High-Beta power and higher T3-2 STAI rating (*r* = 0.456, *p* = 0.019). That is, higher High Beta predicted higher state stress ratings, a result which seems to align with the GAD anxiety rating results.

## Discussion

To our awareness, this is the first study to evaluate the potential anxiety reducing effects of a brief VR intervention specifically designed to help individuals with trait elevated general anxiety. Although both our control and experimental VR intervention groups self-reported decreasing state anxiety across the experiment, significant objective electrophysiological markers associated with reduced anxiety states uniquely appeared only after the VR meditation experience, as opposed to normal periods of rest. The VR meditation resulted in both global and regional decreases in Beta activity. Both effects are in line with electrophysiologically indicating a state of reduced anxiousness. The results of this pilot study provide preliminary evidence supporting that VR interventions may be a useful and effective tool for the treatment of elevated anxiety symptoms. However, the findings and interpretations of these results fall on the background of notable study limitations. Future research is needed to further develop and expand upon the current level of neural mechanistic and processual understanding with regards to how meditative VR simulations affect the brain and mind. As well, further and more robust studies are needed to continue testing the therapeutic efficacy of similar VR meditations on clinical populations, and as compared to other varieties of treatment and control conditions. Despite a number of limitations, discussed in the subsequent sections, this pilot study provides a liminary set of results to build upon as both science and technology continue to advance in this burgeoning area of applied research and intervention.

In this study, both the VR intervention and control participants demonstrated linear decreases in subjective anxiety across all time points. Contrary to our hypotheses, after the VR meditation experience we did not observe a particularly notable reduction in self-reported state anxiety, beyond what the interval of mere quiet rest afforded. Decreases occurred in similar magnitudes irrespective of group or whether the preceding interval contained rest or VR. The ubiquity of this pattern between groups, despite obvious experiential differences between VR and rest, could be due to a number of factors. First, is the possibility that the VR experience was indeed completely comparable to the experience of resting. However, given that the experience of a quiet rest is quite different from the VR immersive experience, it is suspect that the exact same anxiety reducing processes were underway across both intervals for both groups. Furthermore, the current study demonstrated a pattern of spectral changes in the intervention group which differed in significant and important ways from the control group, which indicates that there were indeed unique differences in neurophysiological state brought about by the VR meditative experience.

Alternatively, the observed pattern of STAI responses may have been the result of other potentially strong experimental influences such as characteristic demands (expectations to report reduced anxiety), habituation to the testing environment, psychometric limitations such as floor and ceiling effects, or any combination of these factors. We tend to believe these factors were at least partially influential on the pattern of anxiety rating responses across the testing sessions. While incorporating a control group allowed us to account for and mitigate the confounding effects of some of these considerations (e.g., habituation), we believe that incorporating a variety of anxiety measures in future investigations would help to better delineate the specific magnitude and nature of subjective psychological responses to VR interventions.

Importantly, while both groups showed comparable decreases in self-reported state anxiety, only the VR group, after the VR intervention, uniquely demonstrated additional physiological changes in align with reduced hyperarousal and/or anxiety. Our EEG analysis was focused on Alpha and Beta, and the respective sub-bandwidths’ activity (Alpha1, Alpha2, Low-Beta, High-Beta). The amount of EEG activity occurring across these ranges has been linked with states of relaxation, stress, and anxiety; with increased Alpha being broadly associated with calm and relaxed states, and Beta, particularly higher Beta frequencies, associated with qualitatively anxious states (Thompson and Thompson, 2007; Price and Budzynski, 2009; Olbrich et al., 2011). Our correlational analyses seemed to support such a relationship. GAD scores were significantly inversely correlated with the proportion of Alpha power relative to the full spectrum power profile (i.e., Sum). Moreover, at the sub-band level it was found that lower Alpha frequencies (Alpha1), but not higher Alpha frequencies (Alpha2) supported this relationship. Conversely, High Beta activity significantly predicted higher anxiety scores on both the GAD and the STAI, whereas Low Beta activity did not.

We anticipated some degree of psychological and physiological relaxation response to emerge from a period of quiet rest, but hypothesized that the VR experience would result in unique significant psychophysiological effects beyond experiencing rest alone. Specifically, we hypothesized that we would observe power shifting from higher frequency ranges, such as High-Beta, into lower frequency ranges, such as Alpha. Interestingly, our results tended to indicate power downshifts occurring within (i.e., sub-bands) - but not between - conventional bandwidths (e.g., overall significantly reduced broadband Beta activity coinciding with significantly increased Alpha activity).

Indeed, the pattern of results was much more nuanced. Looking at absolute mean power in Alpha and Beta bandwidths revealed similar power increases across groups, with minor, but not statistically significant divergent patterns when mean power was evaluated within respective sub-bands across the experimental intervals. The observed increased Alpha power for both groups generally fits the narrative of reduced anxiety and increased relaxation, despite no significant extra or unique Alpha enhancement emerging from the VR intervention. Overall, broadband Beta activity demonstrated small but significant increases over time, irrespective of group. However, sub-band dynamics within Beta revealed unique VR effects which supported our general hypotheses.

A more detailed analyses comparing relative power between EEG bands revealed a significant change in the Low-Beta/High-Beta power ratio, specifically occurring in the VR intervention group after the VR experience, but not after rest. While the High Beta frequency range plateaued across the VR interval (and actually decreased relative to total broadband power), the lower Beta range power showed specific increases immediately following the VR exposure. When these shifts in sub-band Beta power were directly compared relative to one another, it was found that the proportion of Low-Beta to High-Beta activity shifted significantly in favor of Low-Beta activity, only for the VR group and only after the VR experience. Although on average the control group had a slightly lower Low-Beta/High-Beta ratio, similar to the VR group, intervals merely involving rest had no effect on the Low-Beta/High-Beta power dynamic.

This finding is significant, indicating a relative reduction of global High-Beta activity only after the VR intervention. This band is of particular interest as it is a the frequency range notably associated with elevated stress and anxiety. In their research, Thompson and Thompson (2007) report that anxiety is generally associated with an increase of High-Beta found in conjunction with a decrease of Low Beta activity; which is the exact opposite of the pattern demonstrated by the VR group in this study. Indeed, our correlational analyses also revealed and support the association between elevated anxiety and High Beta activity. Given our participants were specifically selected based on their elevated anxiety, these shifts in power away from High-Beta are consistent with our hypothesis of reduced anxiety as a result of the VR experience.

Beyond evaluating global electrophysiological pattern changes, we were also interested in select regional activity changes. Using sLORETA, a source localization technique that has been shown to be reasonably effective and accurate, even with relatively low electrode densities such as here (Cohen et al., 1990; Pascual-Marqui et al., 1994; Pascual-Marqui, 1999, 2002, 2007), we examined CSD changes in the anterior and posterior cingulate cortices.

The ACC is thought to serve as a primary mediator between the limbic system and the autonomic nervous system. It is engaged during a range of tasks including decision-making (Rushworth et al., 2007; Morecraft and Tanji, 2009; Shackman et al., 2011), reward processing, conflict monitoring, error detection, and the experience of pain (Vogt, 2005; Beckmann et al., 2009; Shackman et al., 2011). Due to its role in focusing attention, appraisal, and cognitive flexibility, it may come as no surprise that ACC overactivity has also been linked to stress, worry, and cognitive rigidity (Amen, 2001). Other research also suggests ACC overarousal is linked to symptoms of obsessive-compulsive disorder (Rauch et al., 1998; Graybiel and Rauch, 2000). Consistent with this understanding, studies designed to reduce anxiety using respiratory sinus arrhythmia breathing (Sherlin and Wyckoff, 2010) and meditation (Cahn and Polich, 2006; Fox et al., 2016) have demonstrated reduced activation of the ACC. Consistent with these roles and intended goal of anxiety reduction, here we observed a significant decrease of Beta activity in the ACC in the VR but not control group, which occurred only after the VR experience as opposed to an equivalent time of rest. This supports the notion that the VR intervention resulted in changes beyond that experienced by time or quiet rest, and implicates the ACC as one specific region likely affected by the VR meditation, in addition to, or as part of, the global reduction in physiological arousal suggested by our other EEG results.

The other region of interest examined was the PCC. This region is a primary hub of the Default Mode Network (DMN), a group of neural structures strongly linked to self-referencing and other self-related processing (Shulman et al., 1997; Raichle et al., 2001). Recent investigations have identified that the processing of self-related information in the DMN is largely driven by the PCC (Davey et al., 2016). Due to its strong role in self-referencing, research has consistently demonstrated that overarousal of the PCC is connected to a variety of mental health concerns, including ADHD (Nakao et al., 2011), depression (Haznedar et al., 2004), and rumination (Berman et al., 2011). Decreased activity in the PCC is a common finding in meditation literature, consistent with the notion of minimizing self-related thinking (Baerentsen et al., 2010; Fox et al., 2016).

In the current study, we found significant group differences sustained across all intervals, to the effect that PCC activity was generally higher in the VR group compared to controls across Theta, Alpha, and Beta activity ranges. It is unclear why this difference existed. It may somehow be related to the sequential nature of our group data collection, a notable limitation of our study; the precise effects of which are unclear. However, both groups, irrespective of whether the intervals contained rest or the VR intervention, demonstrated similar increases in PCC activation across the duration of the study. Given the broad and non-specific nature of the PCC, it is not entirely clear what this pattern of activity means in terms of contributing to particular psychological or physiological effects. Tentatively, the lack of between group differences suggests that sitting quietly compared to engaging in this type of VR mindfulness meditation for brief periods may have similar impacts on this particular brain region. The PCC has been identified as a neural region involved in self-examination and self-referential processing (Cavanna and Trimble, 2006; Herwig et al., 2012), so it is possible that the changes in the PCC are associated with participants repeatedly self-reflecting on their state anxiety across the course of the study.

Unfortunately, for this pilot, we did not formally assess the content of the subject’s cognitive processes during the experimental or control conditions, instead relying on STAI rating as indicators of cognitive state and stress levels. Despite this limitation, a number of informal observations were made suggesting the VR intervention was having the predicted effect. Some of these observations in experimental group subjects included relaxed shoulder postures and/or slowing of their breathing pattern during the VR experience, which tended to occur between the 2 and 3-min mark of the experience. In addition, several experimental group subjects made spontaneous comments after the VR experience, suggesting that they found it to be enjoyable and/or relaxing. For example, one subject noted “I liked the woman’s voice on the guided meditation. I found it relaxing.”

Summarizing to this point, both groups showed equivalent reductions in state anxiety across time based on subjective ratings. As well, both groups demonstrated increased Alpha power, a common concomitant associated with taking a period of rest. However, a unique effect associated with the VR experience was a global power shift from higher to lower Beta frequencies. Given that prominent high frequency Beta activity is a known marker of state hyperarousal and anxiety, this finding is significant. It tends to support the notion that the VR intervention successfully reduced an important psychophysiological aspect of anxiety, in a way that merely taking a resting break is not able to do.

### Limitations and Future Directions

At the broadest experimental level, are some rather conspicuous limitations. As a pilot evaluation of a newly developed VR meditation intervention, our sample size was small and lacked statistical power to do more robust analyses or detect smaller effects. Future studies evaluating similar interventions on elevated or clinical levels of general anxiety should include larger, possibly more representative samples. Regarding our sample size and composition, here we are somewhat limited in the generalizability of our results. Our sample was comprised of predominantly white women between the ages of 35 and 50. While this may be an important demographic for this type of intervention, the results tell us little about how other populations might respond. Similarly, roughly two-thirds of the sample in this study engaged in some kind of contemplative practice each week. It is unclear how this may have influenced participant interaction with the VR meditation experience. An area that remains to be examined is whether individuals with very different levels of meditative experience engage or benefit from similar VR experiences comparably.

As previously pointed out, another notable limitation was that the two groups, the intervention and the control samples, were recruited and ran separately. Time and resource limitations caused a delay in acquiring the control group sample by about 3 months. The intervention group was fully collected from September-to-November, while the control group was fully collected across February-to-May. It is unclear what, if any, confounding effects resulted from this, suffice to say this was not experimentally optimal and the exact nature and consequence of this serial order, non-randomized, data collection is generally unknown. However, tentatively we did not observe any irrevocable signs of this causing major effects or influences. Our samples were identically recruited, closely matching across demographic characteristics (**Table 1**), and demonstrated very comparable electrophysiological and anxiety reporting patterns across baseline comparisons.

Aside from these more broad-based limitations, we also acknowledge that the results and interpretations of this pilot are by no means definitive, and more research is needed to verify and better elucidate therapeutic mechanisms of this, or similar, VR interventions. Our primary findings were that the VR experience uniquely affected broad and regional Beta activity, with emerging EEG patterns consistent with reductions in anxiety, and physiological hyperarousal. Again, previous research supports our interpretation that this effect is likely indicative of an adaptive state change and a reduction of experiential anxiety; and our results hold early promise based on these connections. However, more research must be conducted to clearly elucidate the nature of high Beta activity in relation to state and trait anxiety manifestations and how interventions, such as we have presented here, can play a maximal therapeutic role. Here, we were restricted in our ability to address such concerns, limited by our reliance on the STAI-state as the sole dependent measure of anxiety. Future research would benefit from including multiple measures to specifically assess changes in cognitive content or somatic indicators of arousal or stress. Our results will need to be replicated and expanded on with a more robust variety of measures.

It will also be important to devise studies which isolate the elements of the VR experience to more clearly define the “active ingredients” of VR based interventions, such as the one tested here. Independently, viewing nature, and practicing mindfulness can both reduce anxiety and promote state changes. This VR experiment has both elements making it unclear if one factor played more of a role that the other, or if perhaps the sum of these parts in the VR experience was/is greater than each factor additively combined. Future studies could also expand on this pilot by including an active control group engaged in a standard relaxation exercise, or an audio-only guided meditation. Such a comparison between groups would provide additional clarity about the potential impact of VR in reducing stress/anxiety above and beyond already existing interventions. Because this is a relatively new and novel approach, future studies with this type of experience should also include follow up questionnaires and/or participant interviews to better ascertain the internal state and reaction to elements of the VR experience, making it easier to interpret specific EEG changes.

Finally, beyond examining the acute effects of a brief single VR session, as we have investigated here, it will also be important to study the effects of longer or repeated VR intervention programs, or to evaluate the potential benefits of VR based meditation experience as an adjunct to traditional psychotherapy. There are still a number of important areas still requiring attention, such as optimal therapeutic VR “dosages,” session frequencies, and/or the very nature of how best to integrate VR interventions into broader treatment plans.

## Conclusion

Our results support the notion that intentionally crafted VR experiences can be therapeutically effective, and may result in immediate, adaptive psychophysiological outcomes. Although there are a number of limitations present in the current study, here we have provided early evidence that VR based meditation interventions have the potential to play an important role in anxiety management and stress reduction. As VR technology becomes more accessible and user-friendly, this type of intervention may find its way into a variety of environments and applications. Because the technology is relatively easy to use, it may serve as a wellness tool in work and school environments, as an intervention for persons with lack of access to nature, as a calming technique for persons receiving medical/dental procedures, as an adjunct to traditional therapeutic interventions, such as CBT programs for GAD, or any number of other applications to increase the personal psychological wellbeing of those in need.

## Datasets are Available on Request

The raw data supporting the conclusions of this manuscript will be made available by the authors, without undue reservation, to any qualified researcher.

## Ethics Statement

The protocol was approved by Quorum Institutional Review Board, Seattle, WA, United States. All subjects gave written informed consent in accordance with the Declaration of Helsinki.

## Author Contributions

JT contributed study conception, design, data collection, and served as the primary writer of the manuscript. JV performed statistical analysis, results reporting, and assisted in manuscript writing. HC organized the database, prepared tables, and assisted in manuscript organization. All authors contributed to manuscript revisions.

## Conflict of Interest Statement

JT is contracted by StoryUp VR to assist in product development and assessment. Coauthor JV, who has no affiliation with StoryUp, was recruited to conduct statistical analyses and assist in the interpretation of this data to reduce potential conflicts of interest. The remaining author declares that the research was conducted in the absence of any commercial or financial relationships that could be construed as a potential conflict of interest.
